# First-in-human Phase I Trial of TPST-1120, an Inhibitor of PPARα, as Monotherapy or in Combination with Nivolumab, in Patients with Advanced Solid Tumors

**DOI:** 10.1158/2767-9764.CRC-24-0082

**Published:** 2024-04-18

**Authors:** Mark Yarchoan, John D. Powderly, Bruno R. Bastos, Thomas B. Karasic, Oxana V. Crysler, Pamela N. Munster, Meredith A. McKean, Leisha A. Emens, Yvonne M. Saenger, Yasser Ged, Robert Stagg, Steven Smith, Chan C. Whiting, Anne Moon, Peppi Prasit, Yonchu Jenkins, Nathan Standifer, Thomas W. Dubensky, Sam H. Whiting, Susanna V. Ulahannan

**Affiliations:** 1 Johns Hopkins Sidney Kimmel Comprehensive Cancer Center, Baltimore, Maryland.; 2 Carolina BioOncology Institute, Huntersville, North Carolina.; 3 Baptist Health Miami Cancer Institute, Miami, Florida.; 4 Abramson Cancer Center at the University of Pennsylvania, Philadelphia, Pennsylvania.; 5 University of Michigan Rogel Cancer Center, Ann Arbor, Michigan.; 6 UCSF Health – UCSF Medical Center, San Francisco, California.; 7 Sarah Cannon Research Institute, Nashville, Tennessee.; 8 UPMC Hillman Cancer Center, Pittsburgh, Pennsylvania.; 9 Herbert Irving Comprehensive Cancer Center, Columbia University, New York, New York.; 10 Tempest Therapeutics, Brisbane, California.; 11 Stephenson Cancer Center of the University of Oklahoma/Sarah Cannon Research Institute, Oklahoma City, Oklahoma.

## Abstract

**Purpose:**

TPST-1120 is a first-in-class oral inhibitor of peroxisome proliferator-activated receptor α (PPARα), a fatty acid ligand-activated transcription factor that regulates genes involved in fatty acid oxidation, angiogenesis, and inflammation, and is a novel target for cancer therapy. TPST-1120 displayed antitumor activity in xenograft models and synergistic tumor reduction in syngeneic tumor models when combined with anti-PD-1 agents.

**Experimental Design:**

This phase I, open-label, dose-escalation study (NCT03829436) evaluated TPST-1120 as monotherapy in patients with advanced solid tumors and in combination with nivolumab in patients with renal cell carcinoma (RCC), cholangiocarcinoma (CCA), or hepatocellular carcinoma. Objectives included evaluation of safety, pharmacokinetics, pharmacodynamics, and preliminary antitumor activity (RECIST v1.1).

**Results:**

A total of 39 patients enrolled with 38 treated (20 monotherapy, 18 combination; median 3 prior lines of therapy). The most common treatment-related adverse events (TRAE) were grade 1–2 nausea, fatigue, and diarrhea. No grade 4–5 TRAEs or dose-limiting toxicities were reported. In the monotherapy group, 53% (10/19) of evaluable patients had a best objective response of stable disease. In the combination group, 3 patients had partial responses, for an objective response rate of 20% (3/15) across all doses and 30% (3/10) at TPST-1120 ≥400 mg twice daily. Responses occurred in 2 patients with RCC, both of whom had previously progressed on anti-PD-1 therapy, and 1 patient with late-line CCA.

**Conclusions:**

TPST-1120 was well tolerated as monotherapy and in combination with nivolumab and the combination showed preliminary evidence of clinical activity in PD-1 inhibitor refractory and immune compromised cancers.

**Significance:**

TPST-1120 is a first-in-class oral inhibitor of PPARα, whose roles in metabolic and immune regulation are implicated in tumor proliferation/survival and inhibition of anticancer immunity. This first-in-human study of TPST-1120 alone and in combination with nivolumab supports proof-of-concept of PPARα inhibition as a target of therapeutic intervention in solid tumors.

## Introduction

Metabolic reprogramming and evasion of immune destruction are hallmarks of cancer (1). While the most widely recognized metabolic adaptation in cancer is an increase in aerobic glycolysis (the Warburg effect), cancer cells can utilize other metabolic pathways such as increased fatty acid oxidation (FAO) to support tumorigenesis. FAO also promotes stemness, drug resistance, and metastasis (2–4), and modulates immune cell function within the tumor microenvironment, which enables tumors to evade antitumor immune responses (5–7). Enhanced FAO is described in multiple cancers and has been reported to correlate with poor patient outcomes (8).

Peroxisome proliferator-activated receptor α (PPARα) is a fatty acid ligand-activated transcription factor that controls the expression of over 100 genes involved in FAO, angiogenesis, and inflammation (9–11). PPARα is critical for maintaining physiologic metabolic homeostasis under conditions when fatty acids are the predominant source of energy, such as during fasting or diet-induced lipid overload. In addition to upregulating genes involved in FAO, activated PPARα dampens Th1-promoting inflammatory responses to metabolic disturbances by directly enhancing transcription of anti-inflammatory proteins, such as IκBα, and by antagonizing the activity of proinflammatory transcription factors, such as NFκB and AP-1, through transrepression, a mechanism involving direct protein–protein interactions (12, 13).

Because of its critical roles in metabolic regulation and immune function, PPARα has emerged as a target of interest for cancer therapy. The dual effect of PPARα both promoting tumor cell growth and inhibiting anticancer immunity is supported by preclinical experiments in which *Ppara* deficiency in either implanted tumor cells or recipient host reduced tumor growth *in vivo*, while *Ppara* deficiency in both implanted tumor cells and recipient host resulted in significantly greater tumor inhibition than in either compartment alone (14). The critical antitumor role of PPARα inhibition specifically in immune cells was further demonstrated in chimeric animals where bone marrow from *Ppara* knockout (KO) mouse transplanted into a *Ppara* wild-type animal, but not the reverse, profoundly inhibited tumor growth (14).

TPST-1120 is a first-in-class, oral, small molecule, competitive antagonist of PPARα, with nanomolar potency (IC_50_ 0.04 µmol/L) for human PPARα and high specificity (>250-fold) for PPARα over the other PPAR isoforms (PPAR β/δ and γ; ref. 15). In xenograft and syngeneic tumor models, TPST-1120 inhibited tumor growth *in vivo* as monotherapy, and the combination of TPST-1120 plus anti-PD-1 therapy resulted in synergistic tumor reduction and durable antitumor immunity in multiple syngeneic mouse models (16). Adoptive transfer of splenocytes from syngeneic mice bearing MC38 colon tumors treated and cured with TPST-1120 plus anti-PD-1 conferred resistance to tumor challenge in naïve mice, similar to the results observed in *Ppara* KO studies (16). This first-in-human study was conducted to evaluate TPST-1120 as monotherapy and in combination with the PD-1 inhibitor nivolumab in patients with select advanced solid tumors.

## Materials and Methods

### Study Design

This was a first-in-human, phase I, open-label, 3+3 design, dose-escalation study (ClinicalTrials.gov identifier: NCT03829436). The primary study objectives were to investigate the safety and tolerability, and to determine the MTD or optimal biological dose (OBD), of TPST-1120 as monotherapy and in combination with nivolumab. Key additional objectives included evaluation of pharmacokinetics, pharmacodynamics, and preliminary assessment of anticancer activity. Exploratory objectives included investigation of immunomodulatory effects of treatment in peripheral blood.

The trial was designed by employees of the study Sponsor, Tempest Therapeutics, in collaboration with the study investigators.

### Patient Selection

Key inclusion criteria included diagnosis of advanced/metastatic solid tumor previously treated with standard systemic therapy for the disease; Eastern Cooperative Oncology Group (ECOG) performance status of 0–1 with estimated life expectancy of at least 12 weeks; adequate organ function; and measurable disease according to the RECIST version 1.1. Tumor types eligible for monotherapy dose escalation [cholangiocarcinoma (CCA), colorectal cancer, metastatic castration-resistant prostate cancer, gastroesophageal cancer, hepatocellular carcinoma (HCC), non–small cell lung cancer, pancreatic cancer, renal cell carcinoma (RCC), sarcoma, head and neck squamous cell carcinoma, triple-negative breast cancer, and urothelial bladder cancer] were selected based upon elevated expression of *PPARA* and associated genes in The Cancer Genome Atlas in these indications. In the nivolumab combination portion of the study, eligible tumor types were limited to those displaying the highest expression of *PPARA* and associated genes: clear cell RCC, CCA, and HCC (16). Key exclusion criteria included history of intolerable or unresolved immune-related adverse event (AE) resulting from prior immune checkpoint inhibitor (ICI) therapy; ongoing use of immunosuppressive medications or active autoimmune disease; untreated/active central nervous system metastases; or use of fibrates within 28 days of enrollment.

### Treatment Plan

Monotherapy TPST-1120 was administered orally in 21-day cycles until disease progression or unacceptable toxicity with a starting dose of 100 mg twice daily (200 mg/day). In a standard 3+3 design, patients were sequentially enrolled at progressively higher dose levels of TPST-1120, evaluating 100, 200, 300, 400, and 600 mg twice daily.

Dose escalation of TPST-1120 in combination with nivolumab was initiated after the 300 mg twice daily (600 mg/day) monotherapy cohort successfully cleared the dose-limiting toxicity (DLT) evaluation period. TPST-1120 was evaluated in 28-day cycles at progressively higher dose levels of 200, 300, 400, and 600 mg orally twice daily in combination with standard dose nivolumab (480 mg intravenous infusion every 4 weeks). Treatment continued until disease progression or unacceptable toxicity.

In all dose cohorts, TPST-1120 was taken with food and with water. Patients were required to fast for a minimum of 8 hours prior to protocol-specified laboratory assessments on cycle 1 day 1, cycle 1 day 8, cycle 2 day 1, and cycle 3 day 1.

### Efficacy and Safety Assessments

Safety was assessed on the basis of incidence of AEs, laboratory results, and physical examinations on day 1 and day 8 of the first cycle, then on day 1 of each subsequent cycle. AEs were recorded by Common Toxicity Criteria for Adverse Events v5.0 from the first dose of study therapy through 28 days after the last dose for monotherapy and 90 days after the last treatment dose for combination therapy.

Disease assessments using CT scans or MRI were performed at baseline and on day 1 of cycle 3, day 1 of cycle 5, and every 9 weeks thereafter for monotherapy, and day 1 of every odd cycle thereafter for combination therapy. Antitumor response was assessed using RECIST v1.1.

### Pharmacokinetics and Pharmacodynamics

Blood for pharmacokinetic and research assessments was collected at screening, cycle 1 days 1, 2, and 8, cycle 2 day 1, cycle 3 day 1, cycle 4 day 1 (combination only), and cycle 5 day 1 (monotherapy only). Tumor biopsies were not required.

Plasma TPST-1120 concentrations were quantified using a validated tandem mass spectrometry assay. Noncompartmental analysis was performed on observed data following the first dose and at steady state on day 8. To assess pharmacodynamic gene expression changes, RNA was extracted from whole blood samples collected in PAXgene Blood RNA tubes on cycle 1 days 1 and 8 and cycle 3 day 1 and assayed on the nCounter instrument (NanoString, Inc.) using the nCounter PanCancer Immune Profiling panel supplemented with an additional 30 PPARα-associated genes (Supplementary Table S1) according to manufacturer's protocols. Associations between gene expression changes and TPST-1120 exposure levels were assessed by linear regression analysis between AUC_0–24_ and baseline-normalized values on day 8, adjusting for the FDR using the method of Benjamini and Hochberg (ref. 17; FDR *P* < 0.05) and demonstrating an effect size greater than 0.5. Similar exposure-related changes in expression had to be observed on cycle 3 day 1 for a gene to be denoted as a pharmacodynamic biomarker. Differences in gene expression change magnitudes between exposure tertiles were assessed using Wilcoxon pairwise method (α = 0.05). To identify associations of gene changes with clinical response, patients were stratified on the basis of best overall response (BOR), and linear discriminant analysis (LDA) was performed to identify gene expression changes on day 8 associated with each response category. Change magnitudes of identified genes were compared between partial responders and stable disease (SD) or progressive disease (PD) patients using Mann–Whitney *U* tests (α = 0.05).

Lipidomics analysis was performed by the UCLA Lipidomics Lab using a LC/MS technique, as described previously (18). Data were analyzed using the method of Su and colleagues (19).

### DLT Definition and OBD Determination

Dose escalation followed a standard 3+3 design with a minimum of 3 patients assigned per dose level. If 0 of 3 or 1 of 6 patients experienced a DLT, dose-escalation continued to the next higher dose level cohort until the MTD was identified or evaluation of the top protocol-defined dose level was completed. Six patients were to be enrolled in the highest dose level cohort that did not exceed the MTD. The MTD was exceeded if >1 of 3 or ≥2 of 6 patients experienced a DLT.

DLTs were evaluated during the first treatment cycle (21 days for monotherapy and 28 days for TPST-1120 plus nivolumab). Patients were evaluable for DLTs if they received at least 85% of planned TPST-1120 doses (and all planned nivolumab doses for combination patients) in the first treatment cycle unless this exposure threshold was not met due to a DLT. Patients who did not meet the DLT evaluability criteria were replaced. AEs occurring during the first treatment cycle and assessed as related to study treatment were considered DLTs following criteria established in the study protocol. OBD determination was based on emerging pharmacokinetics, any pharmacokinetic/pharmacodynamic relationships which could be established, and overall safety and tolerability.

### Statistical Methods

Analysis was performed using descriptive statistics. All patients who received at least one dose of TPST-1120 were included in the safety analysis.

Overall response rate (ORR) was defined as the proportion of patients with complete response or partial response (PR). Disease control rate (DCR) was defined as ORR + SD of at least one scan. The efficacy population (EP) comprised all safety-evaluable patients who had at least one postbaseline tumor assessment, as well as patients who discontinued from study treatment due to PD without undergoing a follow-up radiographic assessment.

### Study Approval

This study was reviewed and approved by the individual site Institutional Review Boards and/or ethics committees where the study was opened and by the FDA. The study was conducted in accordance with the provisions of the Declaration of Helsinki and the International Conference on Harmonization Guidelines for Good Clinical Practice. All patients provided written informed consent.

### Data Availability

The data generated in this study are available upon request from the corresponding author.

## Results

### Patient Characteristics

Between May 13, 2019 and September 07, 2022, a total of 39 patients enrolled at 11 centers in the United States: 21 in the monotherapy cohort and 18 in the combination therapy cohort. One of the 21 patients enrolled in the monotherapy cohort withdrew consent prior to treatment initiation and was not included in the analysis. The baseline characteristics of the study population are presented in Table 1. The mean age was 61.7 years in the monotherapy cohort (range, 41–78 years) and 63.4 years in the combination therapy cohort (range, 43–84 years). In the monotherapy cohort, the primary malignancies were pancreatic cancer [8 (40.0%) patients], CCA [5 (25.0%) patients], and colorectal cancer [4 (20.0%) patients]. In the combination therapy cohort, 9 patients (50.0%) had a primary diagnosis of CCA, while the rest had diagnoses of RCC [5 (27.8%) patients] or HCC [4 (22.2%) patients]. The median number of prior systemic therapies was 3 (range, 2–9) for monotherapy and 3 (range, 1–6) for combination patients. In the combination cohort, all patients with HCC or RCC had received at least one prior anti-PD-(L)1 therapy as part of standard of care and had discontinued the most recent anti-PD-(L)1 therapy for disease progression.

**TABLE 1:** Demographics and patient characteristics of the monotherapy and combination cohorts

Baseline characteristics		TPST-1120 Monotherapy (*n* = 20)	TPST-1120 + Nivolumab (*n* = 18)
Age, mean, years (range)		61.7 (41–78)	63.4 (43–84)
Female, *n* (%)		10 (50)	9 (50)
TPST-1120 dose, *n* (%)	100 mg BID	3 (15)	—
	200 mg BID	4 (20)	3 (17)
	300 mg BID	3 (15)	3 (17)
	400 mg BID	4 (20)	3 (17)
	600 mg BID	6 (30)	9 (50)
Primary cancer type, *n* (%)	Castration-resistant prostate cancer	1 (5)	—
	Cholangiocarcinoma	5 (25)	9 (50)
	Colorectal cancer	4 (20)	—
	Hepatocellular carcinoma	1 (5)	4 (22)
	Non–small cell lung cancer	1 (5)	—
	Pancreatic cancer	8 (40)	—
	Renal cell carcinoma	—	5 (28)
Prior systemic regimens	Median (range)	3 (2–9)	3 (1–6)
	Prior α-PD-1/α-PD-L1, *n* (%)	6 (30)	10 (56)
ECOG PS, *n* (%)	0	5 (25)	8 (44)
	1	15 (75)	10 (56)

Abbreviations: BID, twice daily; ECOG PS, Eastern Cooperative Oncology Group performance status.

### Pharmacokinetics

Data from 31 patients were available for pharmacokinetic analysis on day 8. TPST-1120 steady-state exposure levels increased in a linear, dose-dependent manner and were not affected by nivolumab (Supplementary Fig. S1). Key pharmacokinetic parameters of patients receiving TPST-1120 at 600 mg following single dose and at steady state are listed in Supplementary Table S2.

### Safety

No DLTs occurred during dose escalation. Two monotherapy patients (1 at 200 mg twice daily and 1 at 400 mg twice daily) were not evaluable for DLTs due to missing more than 15% of the required TPST-1120 doses during the evaluation period due to AEs unrelated to treatment and were replaced. An MTD was not established for TPST-1120 as monotherapy or in combination with nivolumab.

In the monotherapy cohort, 10 patients (50%) experienced an AE that was deemed as related to TPST-1120 (Table 2). The most frequently reported treatment-related AEs (TRAE) were nausea [4 (20%) patients], fatigue [3 (15%) patients], and diarrhea [2 (10%) patients], all grade 1–2. One patient experienced grade 3 hypertension at the 600 mg twice daily dose of TPST-1120 that was assessed as treatment-related. There were no grade 4 or grade 5 TRAEs, and no patient discontinued study treatment due to a TRAE.

**TABLE 2:** Treatment-related treatment-emergent AEs by preferred term in ≥1 patient, any grade and grade 3

AE, *n* (%)	Grades 1–3**^a^**	Grade 3
	**TPST-1120 Monotherapy (*n* = 20)**	
Patients with ≥1 TRAE	10 (50.0)	1 (5.0)
Nausea	4 (20.0)	—
Fatigue	3 (15.0)	—
Diarrhea	2 (10.0)	—
Hypertension	1 (5.0)	1 (5.0)
	**TPST-1120 + Nivolumab (*n* = 18)**	
Patients with ≥1 TRAE^b^	14 (77.8)	3 (16.7)
Fatigue	6 (33.3)	—
Diarrhea	4 (22.2)	—
Nausea	3 (16.7)	—
Abdominal pain	2 (11.1)	—
Arthralgia	1 (5.6)	1 (5.6)
Hepatic enzyme increased	1 (5.6)	1 (5.6)
Muscle spasms	1 (5.6)	1 (5.6)

^a^No grade 4 or 5 TRAEs.

^b^Related to either TPST-1120 or nivolumab.

In the combination therapy cohort, 14 patients (77.8%) experienced TRAEs related to either TPST-1120 or nivolumab (Table 2). The most frequent TRAEs were fatigue [6 (33.3%) patients], diarrhea [4 (22%) patients], and nausea [3 (17%) patients], all grade 1–2. Three patients experienced grade 3 TRAEs: 1 each of arthralgia (TPST-1120 400 mg twice daily), hepatic enzymes increased (TPST-1120 600 mg twice daily), and muscle spasms (TPST-1120 600 mg twice daily). Two of these TRAEs (grade 3 arthralgia and grade 3 hepatic enzymes increased) were considered immune-related and treated with systemic steroids. There were no grade 4 or grade 5 TRAEs. The patient with grade 3 hepatic enzymes increased was the sole patient treated with combination therapy (TPST-1120 600 mg orally twice daily) to discontinue treatment due to a TRAE.

### Efficacy

**FIGURE 1:**
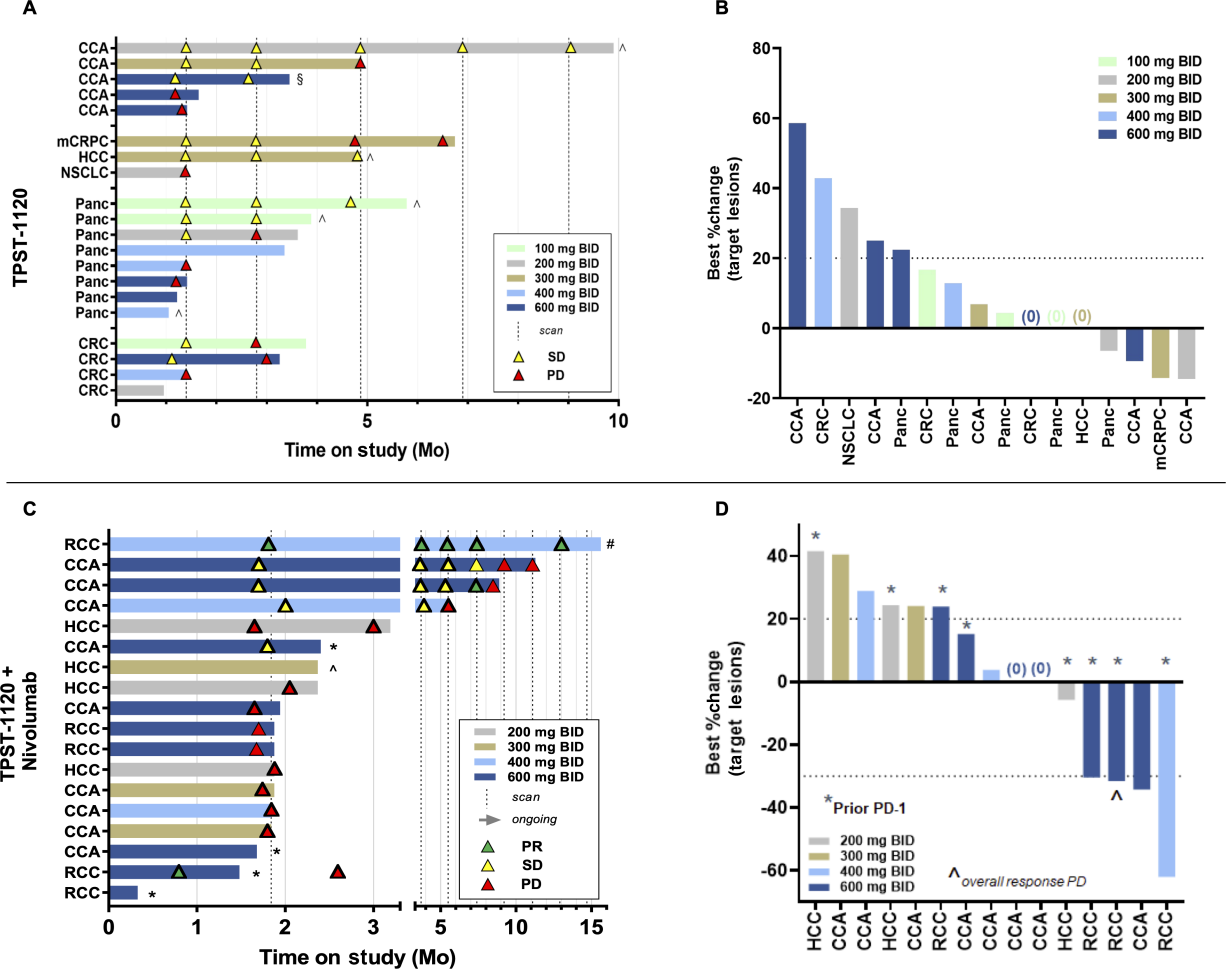
Clinical efficacy of TPST-1120 monotherapy (**A, B**) and in combination with nivolumab (**C, D**). In swimmer plots shown in A and C, study treatment discontinuations for other than disease progression are shown as *Adverse event, ^Symptomatic deterioration, ^#^Investigator decision, or ^§^Consent withdrawn. For monotherapy, scans were every 6 weeks until week 12, followed by an increase to 9-week intervals between scans. For combination therapy, scans occurred every 8 weeks. The RCC responder in the 600 mg BID + nivolumab dose group had an unscheduled scan at day 24 during hospitalization for treatment-unrelated AEs. This patient discontinued treatment one day prior to the scan but was unable to restart and had disease progression during the posttreatment follow-up period. BID, twice daily; CCA, cholangiocarcinoma; CRC, colorectal carcinoma; HCC, hepatocellular carcinoma; mCRPC, metastatic castration-resistant prostate cancer; NSCLC, non–small cell lung cancer; Panc, pancreatic cancer; PD, progressive disease; PR, partial response; RCC, renal cell carcinoma; SD, stable disease.

Clinical efficacy for TPST-1120 monotherapy and in combination with nivolumab is summarized in Fig. 1. Four treated patients were not included in the EP due to discontinuation of treatment for either symptomatic deterioration without objective evidence of PD (1 monotherapy, 1 combination) or due to unrelated AE (2 combination patients) prior to obtaining a postbaseline tumor assessment. The monotherapy efficacy evaluable population included 3 patients who did not have a postbaseline RECIST assessment but discontinued treatment for investigator-assessed disease progression not confirmed by RECIST. Among the 19 efficacy evaluable patients in the monotherapy cohort, the BOR was SD for 10 (53.0%) patients and PD for 6 (32.0%) patients, for a DCR of 53%. Tumor shrinkage of target lesions was observed in 4 patients (21%) with no target lesion growth as the best relative change from baseline in 3 additional patients. Of the 10 patients who had SD, 5 (50%) were on treatment >20 weeks. Among 5 patients with CCA, 3 had at least two assessments of SD, including 1 patient who was on treatment for almost 10 months before discontinuing due to symptomatic deterioration while still meeting RECIST for SD (Fig. 1A; Supplementary Fig. S2).

**FIGURE 2:**
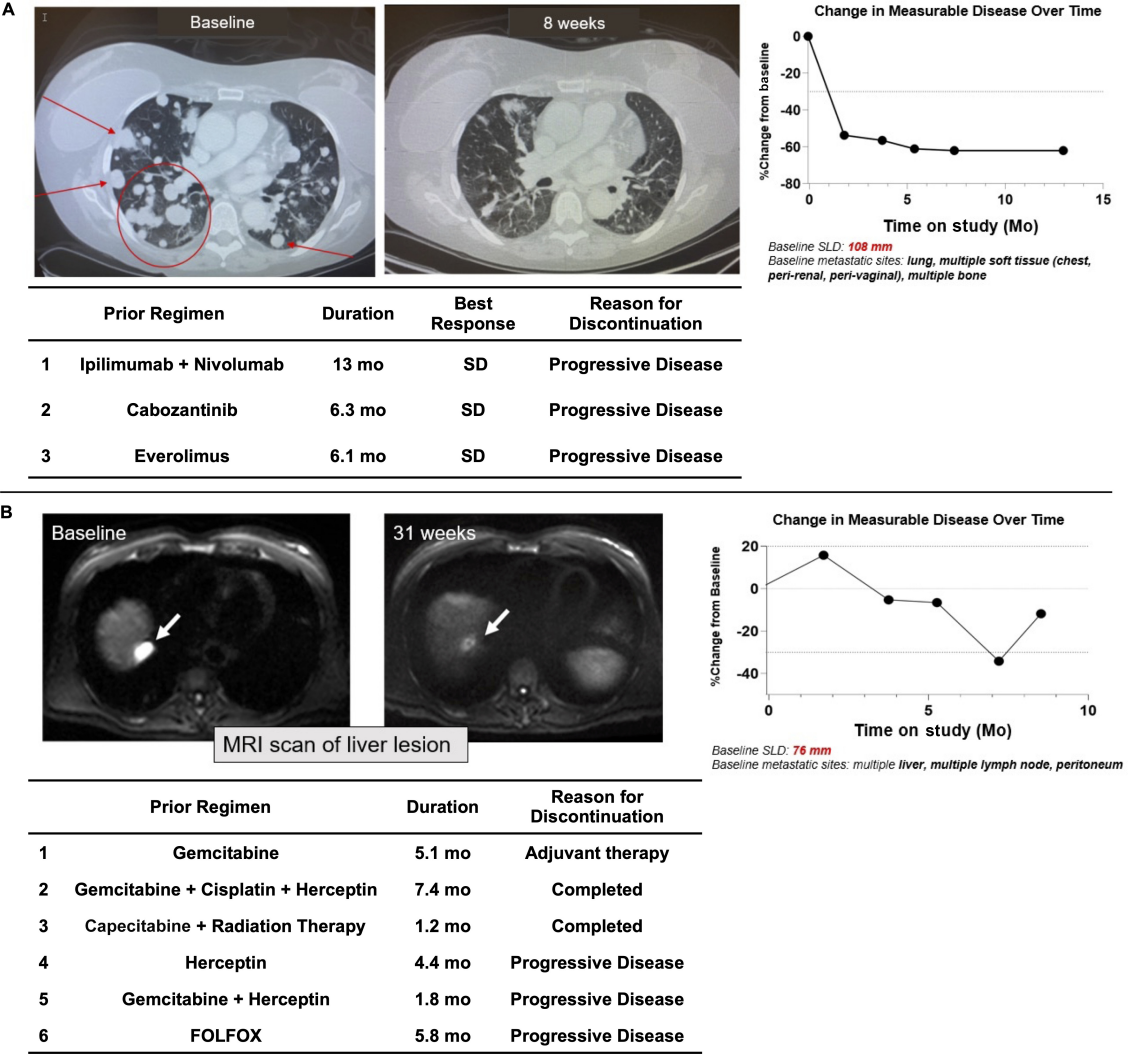
**A,** PR in a 54-year-old female with RCC who had disease progression on prior anti-PD-1 therapy. Prior treatment included ipilimumab + nivolumab (first line), cabozantinib (second line), and everolimus (third line) with no better than SD on any regimen. She achieved a PR (−54%) after 8 weeks of treatment with TPST-1120 + nivolumab that deepened to −62% and was durable for more than 1 year. Changes in pulmonary lesions are shown in CT chest scans taken during screening and on-treatment. SD, stable disease. **B,** PR in an 84-year-old male with heavily pretreated extrahepatic cholangiocarcinoma. He had an initial increase in tumor burden followed by serial shrinking tumor scans before achieving a nadir of −34% and an overall response of PR at scan 4. SLD: sum of longest diameters.

Of the 15 response-evaluable patients in the combination therapy cohort, 3 patients (20.0%) achieved a PR (1 confirmed), while a best response of SD occurred in 3 patients (20%), and 9 patients (60%) had PD. All responders were treated at the two highest doses of TPST-1120 (≥400 mg twice daily) for an ORR of 30% (3/10) at the 400 and 600 mg doses. In addition, of 4 evaluable patients with RCC, 2 achieved a PR for an ORR of 50% in this indication (Fig. 2A for one of the responders) and a third demonstrated a target lesion regression of −31% but also a new lesion in a mixed response (Fig. 1D). One additional PR was achieved in a patient with heavily pretreated CCA that was PD-L1 negative, mismatch repair proficient, with a tumor mutational burden of 10 mut/Mb; he had received six prior lines of systemic therapy before study enrollment (Fig. 2B).

Notably, both patients with RCC who responded to the TPST-1120 combination regimen (and the patient with RCC with a −31% mixed response) were previously treated with at least one anti-PD-1 containing regimen with a best response of SD and discontinued the most recent anti-PD-1 therapy for disease progression. The RCC responder who achieved a −54% RECIST PR at the first tumor assessment (8 weeks of treatment) had previously received first-line ipilimumab + nivolumab followed by cabozantinib and everolimus, discontinuing each regimen for PD after a best response of SD. The RCC responder who achieved a −30% RECIST PR had previously received first-line pembrolizumab + axitinib followed by cabozantinib and discontinued both regimens for disease progression after a best response of SD.

### Biomarker Exploration

**FIGURE 3:**
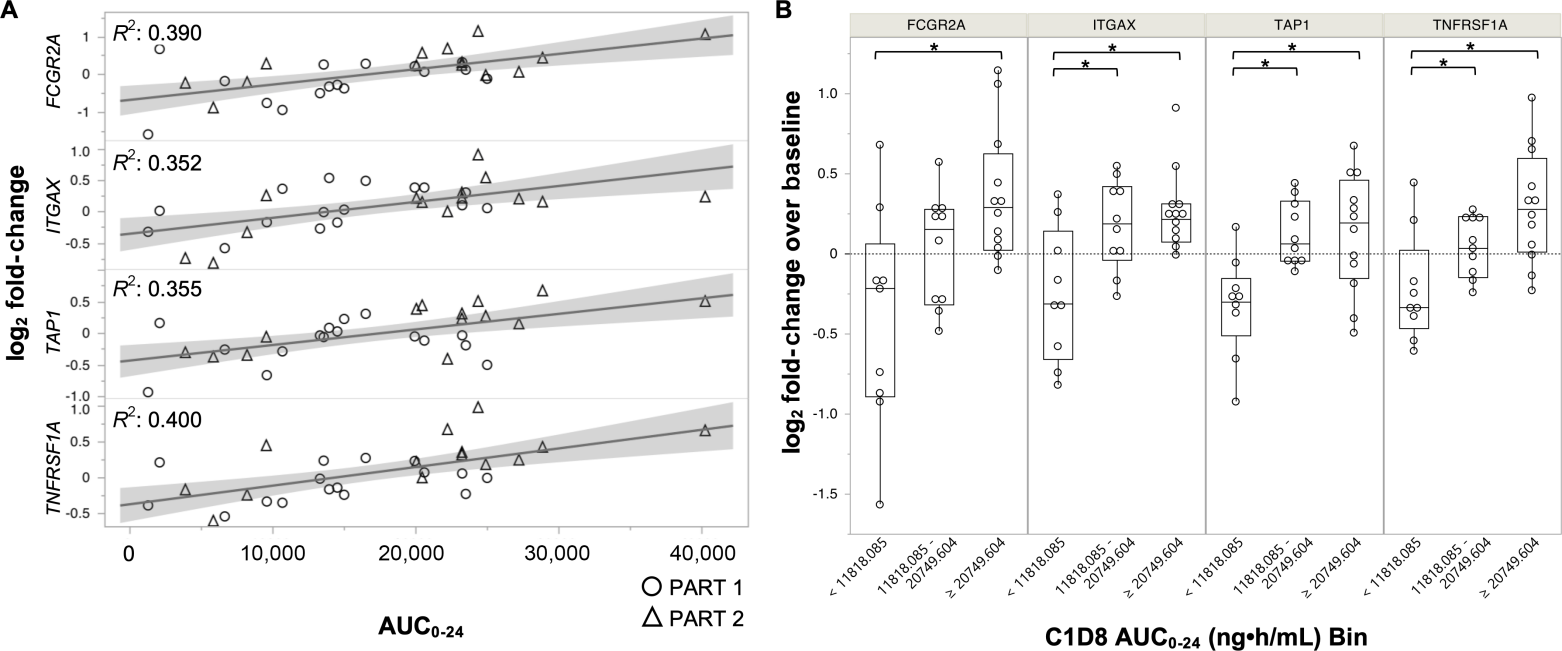
Genes differentially expressed on treatment day 8 versus treatment baseline, as a function of TPST-1120 exposure. **A,** Linear associations of day 8 log_2_ fold change in expression levels of indicated genes and TPST-1120 AUC_0–24_. All genes exhibited FDR *P* < 0.05 and effect size > 0.5. Data are shown for both TPST-1120 monotherapy (part 1) and TPST-1120 + nivolumab combination therapy (part 2). An AUC_0–24_ value of 66,180 ng⋅hour/mL was excluded as an outlier. **B,** Comparison of differential gene expression on day 8 as a function of AUC_0–24_ tertile. Median elevation magnitudes in highest tertile were statistically increased above baseline values (*, *P* < 0.05 by Wilcoxon pair-wise comparison).

Analysis of differential expression levels of 780 genes in whole blood specimens revealed that four were increased as a function of TPST-1120 AUC_0–24_ on day 8 (FDR *P* < 0.05, effect size > 0.5, Fig. 3A). Similar associations between expression levels of these genes and TPST-1120 exposure were also observed on cycle 3 day 1 (Supplementary Fig. S3). Modulated genes included *FCGRIIA* (CD32; ref. 20), *ITGAX* (CD11c; ref. 21), *TAP1* (22, 23), and *TNFRSF1A* (CD120a; ref. 24), all of which are gene targets of transcription factors inhibited by PPARα through transrepression (Table 3). Analysis of gene expression changes by exposure tertile demonstrated that median expression levels of three of the four target genes at the middle exposure tertile, 11,818–20,749 ng⋅hour/mL, were statistically elevated above that observed in the lowest tertile (*P* < 0.05 by Wilcoxon pairwise comparison, Fig. 3B). This corresponds to 60% of patients with TPST-1120 steady-state exposures of at least 11,819 ng⋅hour/mL who demonstrated target gene elevations above baseline levels and identifies a minimum exposure for induction of pharmacodynamic activity.

**TABLE 3:** Summary of genes associated with TPST-1120 exposure levels

Gene	Name	Function in immune cells	FDR *P*-value, effect size	Transcription factor associated with PPARα transrepression
*FCGR2A*	Fc-γ RIIa, CD32	Enhances antibody-dependent cytotoxicity of tumor cells, increased phagocytosis and cytokine release by myeloid cells (25)	0.047, 0.732	STAT1 (20)
*ITGAX*	Integrin α-X, CD11c	Marker of conventional dendritic cells that cross-present tumor antigens, enhances phagocytosis of tumor cells by macrophages (26)	0.049, 0.566	Fos, Jun, C/EBP (21)
*TAP1*	Transporter associated with antigen processing-1	Increases endogenous antigen processing and presentation by MHC-I molecules	0.049, 0.550	STAT1 (22), NFκB (23)
*TNFRSF1A*	TNFα R1, CD120a	Enhances responsiveness to TNFα, activates NFκB-responsive genes (27)	0.047, 0.648	STAT3 (24)

Abbreviation: FDR, false discovery rate.

LDA of day 8 gene expression levels among BOR categories revealed clear distinctions in expression patterns between PD or SD patients and PR patients (Supplementary Fig. S4A). PR patients were observed to express statistically decreased levels of *CFB* and *PVR* (CD155) and increased levels of *APOE*, *MAGEA12*, *RORC,* and *SYT17* (*P* < 0.05 by Mann–Whitney U test; Supplementary Table S3; Supplementary Fig. S4B).

Lipid analysis revealed on-treatment elevation in circulating free fatty acids (FFA) from baseline to day 57 and day 85 in patients demonstrating PRs that were not detected in patients with a best response of PD/SD (Supplementary Fig. S5).

### OBD Selection

The 600 mg twice daily dose of TPST-1120 was determined as the OBD for both monotherapy and combination therapy regimens. Analysis of pharmacokinetics across doses and for both monotherapy and combination showed a linear relationship between TPST-1120 dose and plasma exposure, with no saturation at 600 mg twice daily, the highest dose level tested. Pharmacodynamic analysis of the association between TPST-1120 steady-state exposures and gene expression changes in peripheral blood demonstrated exposure-dependent increases in expression of a subset of genes known to be regulated by transcription factors transrepressed by PPARα, and the identified minimum exposure for induction of pharmacodynamic activity was achieved in all patients receiving TPST-1120 at 400 or 600 mg twice daily (Supplementary Fig. S1). Review of safety data showed no evidence of dose-dependent toxicity through the highest dose tested of 600 mg twice daily, either for single agent TPST-1120 or when administered with nivolumab. Finally, although limited by small numbers, RECIST responses in the combination cohort all occurred at the two highest TPST-1120 dose levels tested, consistent with dose-responsive antitumor activity.

## Discussion

TPST-1120 is a novel investigational agent designed to therapeutically target cancer cells and enhance anticancer immunity by inhibiting the fatty acid ligand-activated transcription factor PPARα. In this phase I first-in-human study, which is the first to report clinical data on PPARα modulation in solid tumors, we tested the hypothesis that inhibiting PPARα with TPST-1120 would be tolerable in patients with advanced cancer and would have anticancer activity. TPST-1120 was well tolerated both as monotherapy and in combination with the PD-1 inhibitor, nivolumab, including no DLTs during dose escalation and predominantly grade 1–2 and manageable TRAEs. In the 18 patients who received TPST-1120 in combination with nivolumab, there was no evidence of synergistic or unexpected toxicity, and the AEs were consistent with the profiles of the two drugs.

TPST-1120 also showed evidence of clinical activity. In the monotherapy group, disease control including target lesion shrinkage was demonstrated in a subset of patients with highly refractory pancreatic, CCA, and late-line colorectal cancers. The combination of TPST-1120 and nivolumab demonstrated an ORR of 20% across all doses and 30% at the two highest doses of TPST-1120. Responses were seen in patients with RCC previously refractory to ICI therapy and in a patient with late-line, PD-L1–negative and microsatellite stable CCA—a tumor type poorly responsive to ICI monotherapy (e.g., pembrolizumab ORR of 2.9% in patients with PD-L1 combined positive score ≤1 treated in the KEYNOTE-158 trial; refs. 28–31). It is notable that among 4 evaluable patients with RCC, 2 (50%) achieved a PR with the TPST-1120 + nivolumab combination, including a deep and very durable response in a patient who had already progressed on nivolumab + ipilimumab after achieving SD as best response. In a recent prospective randomized phase III study, the addition of atezolizumab to cabozantinib provided no additional benefit compared with cabozantinib alone in second-line treatment of patients with RCC who had received ICI treatment in first line (32). In retrospective reports, the ORR of patients with RCC treated with nivolumab monotherapy after previous ICI therapy was 16% (33) and 23% (34). Acknowledging the limitation of small patient numbers, these response data in immunotherapy-refractory patients are consistent with the preclinical results showing that TPST-1120 modulates the immune phenotype away from suppressor populations and combines synergistically with anti-PD-1 therapy (16), and consistent with the literature that genetic KO of PPARα increases inflammation and decreases tumor growth in mouse models (14).

Further supporting the immune mechanism, an exploratory analysis of gene expression changes in whole blood of patients treated in this study demonstrated TPST-1120 exposure-dependent increases in the expression of genes transcriptionally regulated by Th1-promoting proinflammatory transcription factors that are subject to PPARα transrepressive activities, including NFκB and STAT1. This transcriptional dose–response allows for establishment of a minimum exposure threshold for pharmacodynamic activity corresponding to a TPST-1120 dose of at least 400 mg twice daily. Consistent with the exposure threshold, all patients who achieved a PR were dosed with TPST-1120 at ≥400 mg twice daily. Further exploratory analyses of associations between BOR and changes in both gene expression and lipids in study patients showed increases in circulating FFA levels, indicating a reduction in lipid catabolism, and increases in *RORC*, encoding RORγt, the master transcriptional regulator of Th17 cells, consistent with literature reports that Th17 cells are increased in PPARα-deficient animals (35, 36).

The increased expression of genes downstream of proinflammatory transcription factor targets of PPARα transrepression suggests that immune cell function is enhanced with TPST-1120 treatment. These results are consistent with literature indicating PPARα deficiency increases inflammation, one such example being the promotion of M1 macrophage polarization by *Ppara* KO in myeloid cells—shown as increased IL1 and TNFα mRNA and decreased arginase mRNA expression upon lipopolysaccharide stimulation of bone marrow–derived macrophages (37). Lack of PPARα, as well as treatment with the PPARα antagonist IS001, resulted in increased IFNγ production by CD4^+^ and CD8^+^ T lymphocytes and natural killer T cells (38). Correspondingly, augmented or sustained responses to inflammatory stimuli across multiple models of inflammation were observed when *Ppara* was deleted and support a role for PPARα in immune regulation (39–41).

In characterizing the antitumor activity of TPST-1120, it is of interest whether these dual PPARα functions—transrepression associated with immunomodulatory activity and PPAR response element (PPRE)-mediated transcription controlling lipid metabolism—are linked, as increased utilization of FAO is a well-studied metabolic characteristic of suppressive immune cells. Experiments conducted using a mutant PPARα protein unable to activate transcription of PPRE-dependent genes and consequently devoid of its lipid-regulating activity, showed that mutant PPARα retained the ability to attenuate inflammation in a mouse model of liver fibrosis, demonstrating that these two functions can be uncoupled and are distinct (42). Future preclinical work will focus on dissecting the contribution of each function to the antitumor activity displayed by TPST-1120.

Limitations of this study include small numbers of patients treated at different TPST-1120 dose levels. In addition, imbalanced enrollment of pancreaticobiliary cancers in the monotherapy cohort reduced the assessment of the PPARα inhibition mechanism in other solid tumor types. Evaluation of TPST-1120 pharmacodynamic activity was restricted to the challenging and complex setting of peripheral blood cells and circulating lipids by the lack of fresh tumor biopsies.

Further clinical development of TPST-1120 in RCC and CCA is planned based upon these study results. A separate study is ongoing in HCC, a tumor type not adequately represented in this phase I study, but of particular interest due to the high expression of PPARα. A global randomized phase Ib/II study is evaluating the combination of atezolizumab plus bevacizumab, with or without TPST-1120, in patients with first-line advanced HCC (NCT04524871). Initial efficacy results from this randomized study are encouraging and have been publicly reported (43). The identification of novel biomarkers that correlate with sensitivity to TPST-1120 may further improve patient selection for future trials and inform the development of novel therapeutic combinations.

## Supplementary Material

Supplementary Table S1 Additional PPAR-α Associated Genes Monitored

Supplementary Table S2 Summary of pharmacokinetics of 600 mg TPST-1120 twice daily as a single agent and in combination with nivolumab after single dose or at steady state (cycle 1 day 8)

Supplementary Table S3 Summary of Differentially Expressed Genes in PR Patients (p-value < 0.05 by Mann-Whitney U-test vs. PR/SD patients) on Cycle 1 Day 8

Supplementary Figure S1 Relationship between dose (mg BID) and AUC0-24 of TPST-1120 at steady state on Cycle 1 Day 8. Monotherapy and nivolumab combination data points offset slightly for clarity.

Supplementary Figure S2 Monotherapy tumor control in late-line cholangiocarcinoma. Change in measurable tumor burden over time is shown for two patients with late-line cholangiocarcinoma demonstrating prolonged disease control achieved with monotherapy TPST-1120, including patient B who achieved multiple stable disease scans with serial shrinkage of tumor burden to a nadir of -13% by RECIST over a duration of 9.5 months on treatment. Prior systemic treatment for patient A included cisplatin/gemcitabine, an investigational multi-kinase inhibitor, and an investigational anti-PD-1, while patient B received carboplatin/taxol, gemcitabine, oxaliplatin/capecitabine, and an investigational anti-PD-1/indoleamine 2,3-dioxygenase 1 inhibitor combination. Both patients discontinued the most recent therapy regimen received prior to TPST-1120 treatment for progressive disease.

Supplementary Figure S3 Genes differentially expressed as a function of BOR on cycle 3 day 1. Genes differentially expressed on C3D1 versus treatment baseline, as a function of TPST-1120 exposure. Linear associations of C3D1 Log2 fold change in expression levels of indicated genes and TPST-1120 AUC0-24. Data are shown for both TPST-1120 monotherapy (part 1) and TPST-1120 + nivolumab combination therapy (part 2).

Supplementary Figure S4 Genes differentially expressed as a function of BOR on day 8. (A) Linear discriminant analysis of Log2 fold change in expression levels of 780 genes in patients stratified based on BOR. PR: partial response; SD: stable disease; PD: progressive disease. (B) Genes that maximally discriminate between PR and PD patients enrolled in combination therapy arm. * p<0.05 by Mann-Whitney U-test.

Supplementary Figure S5 Elevated circulating free fatty acids (FFA) at days 57 and 85 in PR patients. Log2-fold changes in baseline normalized FFA in patients enrolled in Part 2. Samples were collected prior to TPST-1120 dose administration on day 1 of each 28-day cycle. Blue symbols: PD/SD patients; Red symbols: PR patients.
