# Supplementary material for: First-in-human Phase I Trial of TPST-1120, an Inhibitor of PPARα, as Monotherapy or in Combination with Nivolumab, in Patients with Advanced Solid Tumors
Source: Cancer Res Commun. 2024 Apr 18;4(4):1100–10. doi: 10.1158/2767-9764.CRC-24-0082 (PMC11025498; doi:10.1158/2767-9764.CRC-24-0082)
Supplement: Supplementary Table S1 — Additional PPAR-α Associated Genes Monitored [file crc-24-0082-s01.pdf]

Supplementary Table S1. Additional PPAR-α Associated Genes Monitored

HGNC Gene Symbol	Gene Name
ACAA2	acetyl-CoA acyltransferase 2
ACAD8	acyl-CoA dehydrogenase family member 8
ACOX1	acyl-CoA oxidase 1
ACSL3	acyl-CoA synthetase long chain family member 3
ACSL5	acyl-CoA synthetase long chain family member 5
APOA5	apolipoprotein A5 (APOA-V)
CPT1A	carnitine palmitoyltransferase 1A
CPT1B	carnitine palmitoyltransferase 1B
CPT2	carnitine palmitoyltransferase 2
CYP1A1	cytochrome P450 family 1 subfamily A member 1
CYP4A11	cytochrome P450 family 4 subfamily A member 11
FABP1	fatty acid binding protein 1
FABP3	fatty acid binding protein 3
FABP4	fatty acid binding protein 4
FABP5	fatty acid binding protein 5
FADS2	acyl-CoA 6-desaturase
FASN	fatty acid synthase
FGF21	fibroblast growth factor 21
HADHA	hydroxyacyl-CoA dehydrogenase trifunctional multienzyme complex subunit alpha
HADHB	hydroxyacyl-CoA dehydrogenase trifunctional multienzyme complex subunit beta
HMGCS2	3-hydroxy-3-methylglutaryl-CoA synthase 2
MT1A	Metallothionein 1A
PANK1	pantothenate kinase 1
PDK4	pyruvate dehydrogenase kinase 4
PLIN1	perilipin 1
PPARA	peroxisome proliferator activated receptor alpha
PPARD	peroxisome proliferator activated receptor delta
PPARGC1A	PPARG coactivator 1 alpha
SLC25A20	solute carrier family 25 member 20
VNN1	vanin 1
