# Supplementary material for: First-in-human Phase I Trial of TPST-1120, an Inhibitor of PPARα, as Monotherapy or in Combination with Nivolumab, in Patients with Advanced Solid Tumors
Source: Cancer Res Commun. 2024 Apr 18;4(4):1100–10. doi: 10.1158/2767-9764.CRC-24-0082 (PMC11025498; doi:10.1158/2767-9764.CRC-24-0082)
Supplement: Supplementary Table S2 — Summary of pharmacokinetics of 600 mg TPST-1120 twice daily as a single agent and in combination with nivolumab after single dose or at steady state (cycle 1 day 8) [file crc-24-0082-s02.pdf]

Supplementary Table S2. Summary of pharmacokinetics of 600 mg TPST-1120 twice daily as a single agent and in combination with nivolumab after single dose or at steady state (cycle 1 day 8)

	**600 mg BID Monotherapy + Combination**	
**Parameter**	**n**	**Mean (CV%)**
**Single dose**		
$AUC_{0-\tau}$ (ng*h/mL)	15	12,784.7 (58.7)
C_{max} (ng/mL)	15	3,634.7 (52.8)
T_{max} (h)	15	2[a]
Half-life (h)	11	4.25 (98.4)
CL/F (L/h)	11	46.44 (56.6)
Vz/F (L)	11	296.5 (154.2)
**Steady state**		
AUC_{0-12} (ng*h/mL)	13	13,444.6 (49.9)
C_{max} (ng/mL)	13	3,675.4 (47.9)
T_{max} (h)	13	1[a]
CL/F (L/h)	13	51.4 (32.6)
R_{ac} (AUC)	13	1.08

Abbreviations: AUC_{0-T}, area under the concentration time curve, from time 0 to the last time point with a quantifiable plasma concentration (C1D1 single dose only); AUC_{0-12}, area under the concentration time curve from time 0 to 12 hours; C_{max}, maximum observed concentration in plasma; CL/F, apparent oral clearance; CV, coefficient of variation; R_{ac}, Ratio of accumulation of drug at steady state compared to single dose; T_{max}, time of maximum observed concentration in plasma; Vz/F, apparent oral volume of distribution.

[a]Median value.
