# Supplementary material for: First-in-human Phase I Trial of TPST-1120, an Inhibitor of PPARα, as Monotherapy or in Combination with Nivolumab, in Patients with Advanced Solid Tumors
Source: Cancer Res Commun. 2024 Apr 18;4(4):1100–10. doi: 10.1158/2767-9764.CRC-24-0082 (PMC11025498; doi:10.1158/2767-9764.CRC-24-0082)
Supplement: Supplementary Table S3 — Summary of Differentially Expressed Genes in PR Patients (p-value < 0.05 by Mann-Whitney U-test vs. PR/SD patients) on Cycle 1 Day 8 [file crc-24-0082-s03.pdf]

Supplementary Table S3. Summary of Differentially Expressed Genes in PR Patients (p-value < 0.05 by Mann-Whitney U-test vs. PR/SD patients) on Cycle 1 Day 8

**Gene**	**Name**	**Function**	**Differential Expression in PR patients**
RORC	RAR related Orphan Receptor C	Transcriptional regulator of Th17 cell differentiation	Increased
APOE	Apolipoprotein E	Lipid metabolism	Increased
MAGEA12	Melanoma-associated Antigen-12	Repressor of tumor-suppressor genes	Increased
SYT17	Synaptotagmin 17	Intracellular membrane trafficking	Increased
PVR	Poliovirus Receptor, CD155	TIGIT ligand	Decreased
CFB	Complement Factor B	Alternative complement pathway, C3B convertase	Decreased
