# Supplementary material for: First-in-human Phase I Trial of TPST-1120, an Inhibitor of PPARα, as Monotherapy or in Combination with Nivolumab, in Patients with Advanced Solid Tumors
Source: Cancer Res Commun. 2024 Apr 18;4(4):1100–10. doi: 10.1158/2767-9764.CRC-24-0082 (PMC11025498; doi:10.1158/2767-9764.CRC-24-0082)
Supplement: Supplementary Figure S1 — Relationship between dose (mg BID) and AUC0-24 of TPST-1120 at steady state on Cycle 1 Day 8. Monotherapy and nivolumab combination data points offset slightly for clarity. [file crc-24-0082-s04.pdf]

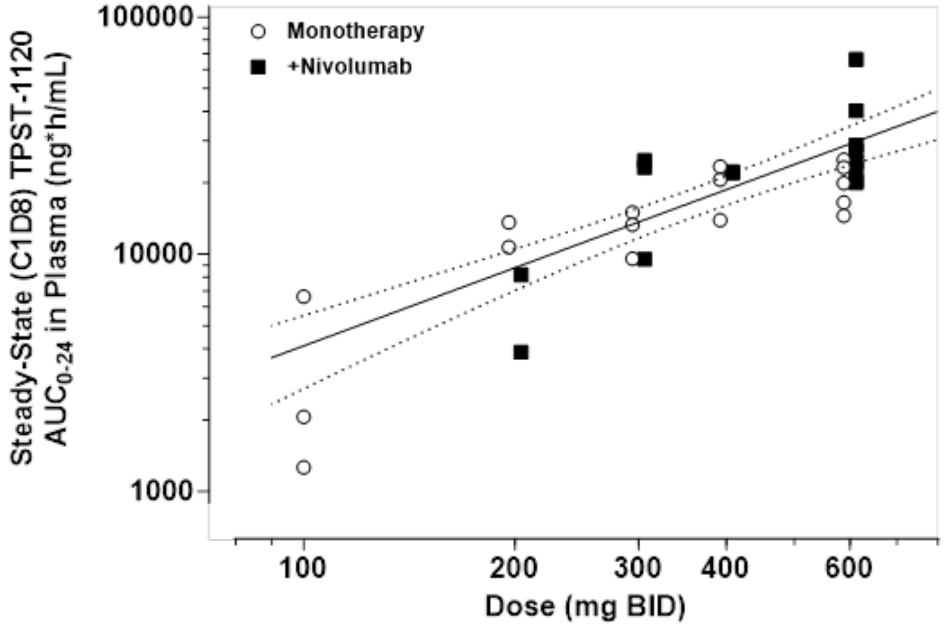

Supplementary Figure S1. Relationship between dose (mg BID) and $AUC_{0\text{-}24}$ of TPST-1120 at steady state on Cycle 1 Day 8. Monotherapy and nivolumab combination data points offset slightly for clarity.
