# Supplementary material for: First-in-human Phase I Trial of TPST-1120, an Inhibitor of PPARα, as Monotherapy or in Combination with Nivolumab, in Patients with Advanced Solid Tumors
Source: Cancer Res Commun. 2024 Apr 18;4(4):1100–10. doi: 10.1158/2767-9764.CRC-24-0082 (PMC11025498; doi:10.1158/2767-9764.CRC-24-0082)
Supplement: Supplementary Figure S2 — Monotherapy tumor control in late-line cholangiocarcinoma. Change in measurable tumor burden over time is shown for two patients with late-line cholangiocarcinoma demonstrating prolonged disease control achieved with monotherapy TPST-1120, including patient B who achieved multiple stable disease scans with serial shrinkage of tumor burden to a nadir of -13% by RECIST over a duration of 9.5 months on treatment. Prior systemic treatment for patient A included cisplatin/gemcitabine, an investigational multi-kinase inhibitor, and an investigational anti-PD-1, while patient B received carboplatin/taxol, gemcitabine, oxaliplatin/capecitabine, and an investigational anti-PD-1/indoleamine 2,3-dioxygenase 1 inhibitor combination. Both patients discontinued the most recent therapy regimen received prior to TPST-1120 treatment for progressive disease. [file crc-24-0082-s05.pdf]

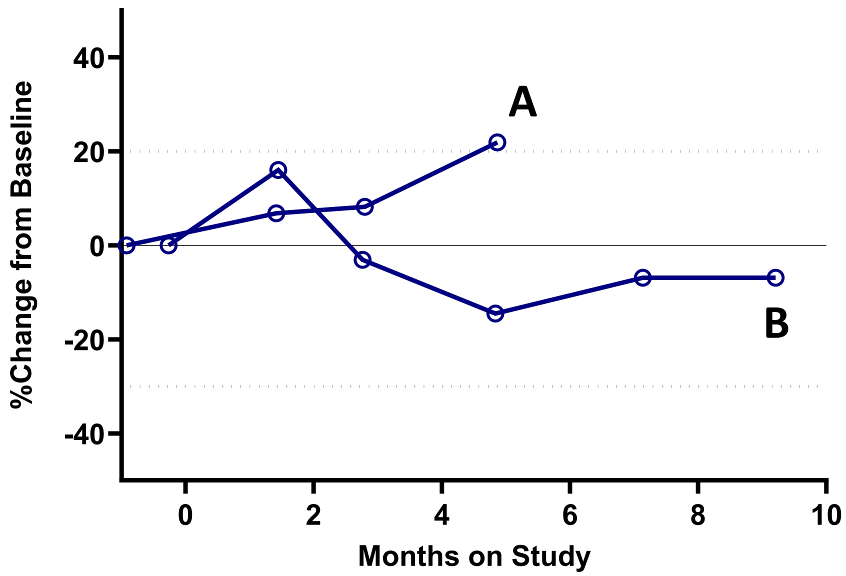

Supplementary Figure S2. Monotherapy tumor control in late-line cholangiocarcinoma. Change in measurable tumor burden over time is shown for two patients with late-line cholangiocarcinoma demonstrating prolonged disease control achieved with monotherapy TPST-1120, including patient B who achieved multiple stable disease scans with serial shrinkage of tumor burden to a nadir of -13% by RECIST over a duration of 9.5 months on treatment. Prior systemic treatment for patient A included cisplatin/gemcitabine, an investigational multi-kinase inhibitor, and an investigational anti-PD-1, while patient B received carboplatin/taxol, gemcitabine, oxaliplatin/capecitabine, and an investigational anti-PD-1/indoleamine 2,3-dioxygenase 1 inhibitor combination. Both patients discontinued the most recent therapy regimen received prior to TPST-1120 treatment for progressive disease.
