# Supplementary material for: First-in-human Phase I Trial of TPST-1120, an Inhibitor of PPARα, as Monotherapy or in Combination with Nivolumab, in Patients with Advanced Solid Tumors
Source: Cancer Res Commun. 2024 Apr 18;4(4):1100–10. doi: 10.1158/2767-9764.CRC-24-0082 (PMC11025498; doi:10.1158/2767-9764.CRC-24-0082)
Supplement: Supplementary Figure S3 — Genes differentially expressed as a function of BOR on cycle 3 day 1. Genes differentially expressed on C3D1 versus treatment baseline, as a function of TPST-1120 exposure. Linear associations of C3D1 Log2 fold change in expression levels of indicated genes and TPST-1120 AUC0-24. Data are shown for both TPST-1120 monotherapy (part 1) and TPST-1120 + nivolumab combination therapy (part 2). [file crc-24-0082-s06.pdf]

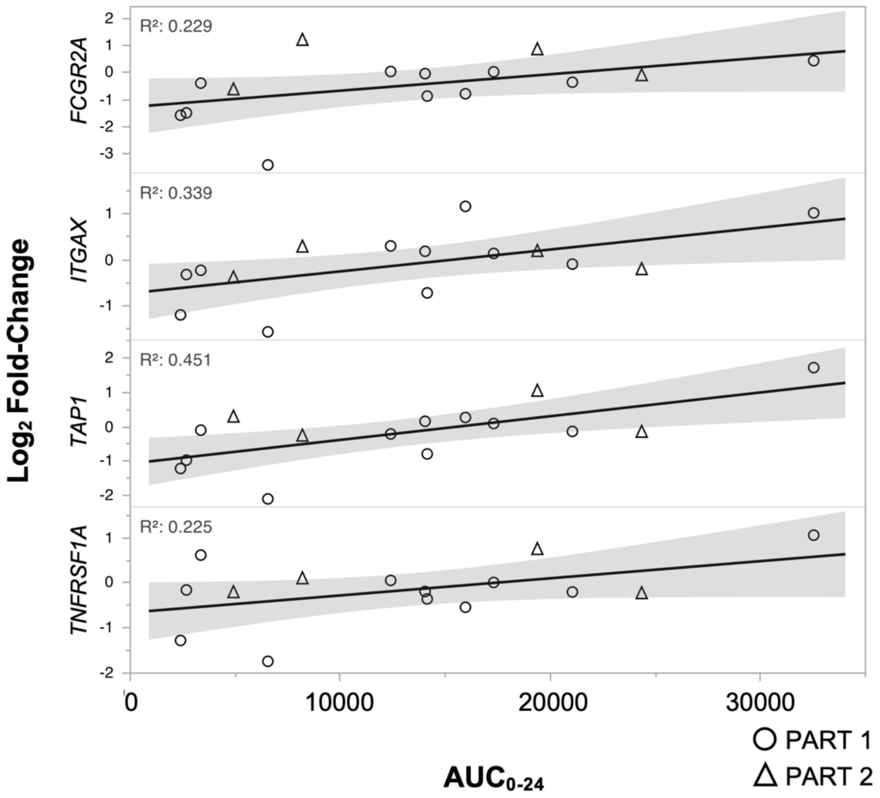

Supplementary Figure S3. Genes differentially expressed as a function of BOR on cycle 3 day 1. Genes differentially expressed on C3D1 versus treatment baseline, as a function of TPST-1120 exposure. Linear associations of C3D1 Log_2 fold change in expression levels of indicated genes and TPST-1120 $AUC_{0\text{-}24}$. Data are shown for both TPST-1120 monotherapy (part 1) and TPST-1120 + nivolumab combination therapy (part 2).
