# Supplementary material for: First-in-human Phase I Trial of TPST-1120, an Inhibitor of PPARα, as Monotherapy or in Combination with Nivolumab, in Patients with Advanced Solid Tumors
Source: Cancer Res Commun. 2024 Apr 18;4(4):1100–10. doi: 10.1158/2767-9764.CRC-24-0082 (PMC11025498; doi:10.1158/2767-9764.CRC-24-0082)
Supplement: Supplementary Figure S4 — Genes differentially expressed as a function of BOR on day 8. (A) Linear discriminant analysis of Log2 fold change in expression levels of 780 genes in patients stratified based on BOR. PR: partial response; SD: stable disease; PD: progressive disease. (B) Genes that maximally discriminate between PR and PD patients enrolled in combination therapy arm. * p<0.05 by Mann-Whitney U-test. [file crc-24-0082-s07.pdf]

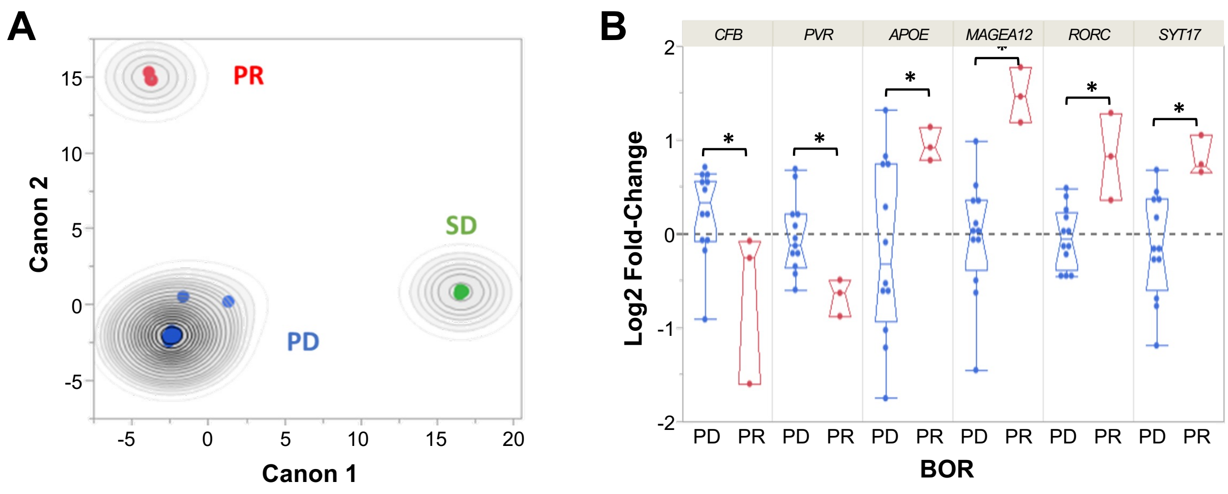

Supplementary Figure S4. Genes differentially expressed as a function of BOR on day 8. (A) Linear discriminant analysis of Log_2 fold change in expression levels of 780 genes in patients stratified based on BOR. PR: partial response; SD: stable disease; PD: progressive disease. (B) Genes that maximally discriminate between PR and PD patients enrolled in combination therapy arm. * p<0.05 by Mann-Whitney U-test.
