# Supplementary material for: First-in-human Phase I Trial of TPST-1120, an Inhibitor of PPARα, as Monotherapy or in Combination with Nivolumab, in Patients with Advanced Solid Tumors
Source: Cancer Res Commun. 2024 Apr 18;4(4):1100–10. doi: 10.1158/2767-9764.CRC-24-0082 (PMC11025498; doi:10.1158/2767-9764.CRC-24-0082)
Supplement: Supplementary Figure S5 — Elevated circulating free fatty acids (FFA) at days 57 and 85 in PR patients. Log2-fold changes in baseline normalized FFA in patients enrolled in Part 2. Samples were collected prior to TPST-1120 dose administration on day 1 of each 28-day cycle. Blue symbols: PD/SD patients; Red symbols: PR patients. [file crc-24-0082-s08.pdf]

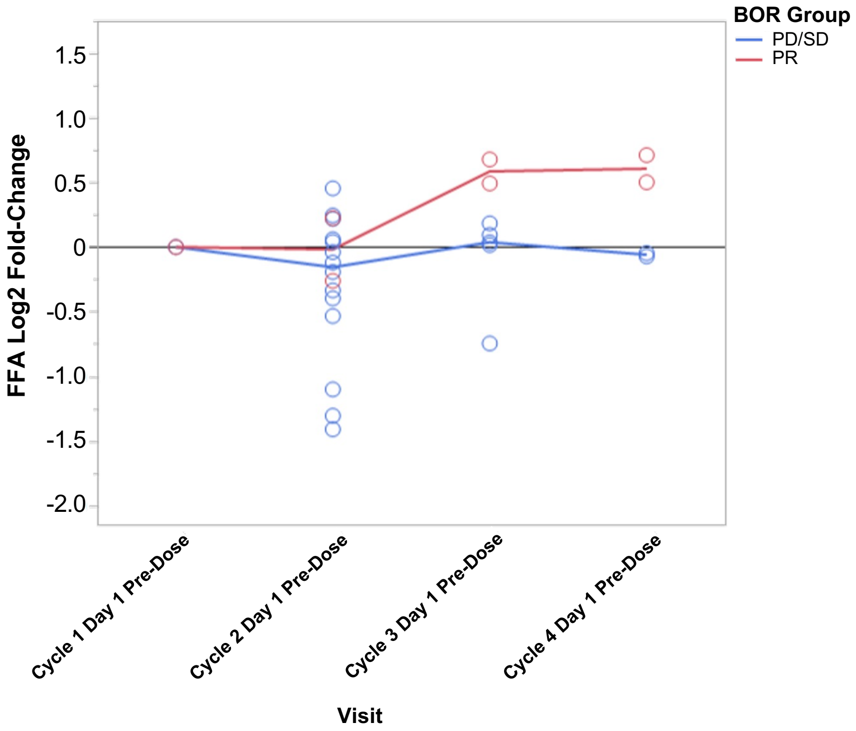

Supplementary Figure S5. Elevated circulating free fatty acids (FFA) at days 57 and 85 in PR patients. Log_2-fold changes in baseline normalized FFA in patients enrolled in Part 2. Samples were collected prior to TPST-1120 dose administration on day 1 of each 28-day cycle. Blue symbols: PD/SD patients; Red symbols: PR patients.
